# Outer membrane vesicles derived from *Bordetella pertussis* are potent adjuvant that drive Th1-biased response

**DOI:** 10.3389/fimmu.2024.1387534

**Published:** 2024-04-08

**Authors:** Bernarda Pschunder, Lucia Locati, Oriana López, Pablo Martin Aispuro, Eugenia Zurita, Matthew Stuible, Yves Durocher, Daniela Hozbor

**Affiliations:** ^1^ Laboratorio Vacunas Salud (VacSal), Instituto de Biotecnología y Biología Molecular (IBBM), Facultad de Ciencias Exactas, Universidad Nacional de La Plata, Centro Científico Tecnológico-Consejo Nacional de Investigaciones Científicas y Técnicas (CCT-CONICET) La Plata, La Plata, Argentina; ^2^ Human Health Therapeutics Research Center, National Research Council Canada, Montreal, QC, Canada

**Keywords:** *Bordetella pertussis*, outer-membrane vesicles, adjuvant, Th1, antibodies, alum

## Abstract

For several years, we have been committed to exploring the potential of *Bordetella pertussis*-derived outer membrane vesicles (OMV*_Bp_*) as a promising third-generation vaccine against the reemerging pertussis disease. The results of our preclinical trials not only confirm its protective capacity against *B. pertussis* infection but also set the stage for forthcoming human clinical trials. This study delves into the examination of OMV*_Bp_* as an adjuvant. To accomplish this objective, we implemented a two-dose murine schedule to evaluate the specific immune response induced by formulations containing OMV*_Bp_* combined with 3 heterologous immunogens: Tetanus toxoid (T), Diphtheria toxoid (D), and the SARS-CoV-2 Spike protein (S). The specific levels of IgG, IgG1, and IgG2a triggered by the different tested formulations were evaluated using ELISA in dose-response assays for OMV*_Bp_* and the immunogens at varying levels. These assays demonstrated that OMV*_Bp_* exhibits adjuvant properties even at the low concentration employed (1.5 μg of protein per dose). As this effect was notably enhanced at medium (3 μg) and high concentrations (6 μg), we chose the medium concentration to determine the minimum immunogen dose at which the OMV adjuvant properties are significantly evident. These assays demonstrated that OMV*_Bp_* exhibits adjuvant properties even at the lowest concentration tested for each immunogen. In the presence of OMV*_Bp_*, specific IgG levels detected for the lowest amount of antigen tested increased by 2.5 to 10 fold compared to those found in animals immunized with formulations containing adjuvant-free antigens (p<0.0001). When assessing the adjuvant properties of OMV*_Bp_* compared to the widely recognized adjuvant alum, we detected similar levels of specific IgG against D, T and S for both adjuvants. Experiments with OMVs derived from *E. coli* (OMV*_E.coli_*) reaffirmed that the adjuvant properties of OMVs extend across different bacterial species. Nonetheless, it’s crucial to highlight that OMV*_Bp_* notably skewed the immune response towards a Th1 profile (p<0.05). These collective findings emphasize the dual role of OMV*_Bp_* as both an adjuvant and modulator of the immune response, positioning it favorably for incorporation into combined vaccine formulations.

## Introduction

Since the discovery of outer membrane vesicles (OMVs) and their initial detection in *Vibrio cholerae* through microscopy in 1967 by Chatterjee and Das (1), evidence of their production by various Gram-negative bacteria and their multiple roles in host-pathogen interactions has accumulated over the years (2–6). In the past decade, numerous studies have unveiled the functions of OMVs in bacterial physiology and pathogenesis, the delivery of virulence factors to host cells, and their role in enhancing bacterial survival in the community and adverse environments (2, 3, 7–9). Due to their composition, which includes bacteria-derived antigens and a variety of pathogen-associated molecular patterns (PAMPs) in their natural state, OMVs are recognized by host Toll-like receptors (e.g., TLR4 and TLR2), as well as the non-canonical inflammasome via caspase-11 (10, 11). Additionally, their size ranges from 20 to 250 nm, facilitating efficient uptake by antigen-presenting cells (APCs) and triggering a robust immune response. As a result, both natural and bioengineered OMVs have emerged as promising objects for biomedical applications. Particularly, they are being investigated as bacterial vaccines aiming to elicit strong immune responses at both humoral (antibodies) and cellular levels (immune cells and cytokines) (3, 7, 12–14). The first vaccine approved for human use containing OMVs is targets infections caused by *Neisseria meningitidis* serogroup B (12, 15, 16). Additionally, OMVs have been proposed and evaluated in preclinical trials by our group initially and subsequently by others as vaccines to better control pertussis (14, 17–19), a resurgent respiratory disease (20–23). The rationale behind this proposed vaccine candidate is that many of the known virulence factors and immunogens are components of the outer membrane of the disease-causing agent *Bordetella pertussis* (5, 24). Using the murine intranasal challenge model we have shown that OMVs are not only potent immunogens that induce a response impacting protection against bacterial infection, specifically a Th1 response profile with memory response in respiratory tissue, but they have also proven to be less reactogenic than the current commercially available vaccine based on inactivated detoxified bacterial cells (17, 18, 24–26). Compared to the other commercially available vaccine, the commercial acellular vaccines, the OMV-based formulation has demonstrated superiority not only in the type of immune response induced but also in its effectiveness in protecting against *B. pertussis* clinical isolates that are currently more resistant to existing commercial vaccines (26). Interesting finding was also obtained with the OMVs derived from *B. parapertussis* which exhibited in the accepted animal model a cross-protection against infections caused by both *B. pertussis* and *B. parapertussis* (27).

For a number of other human pathogens OMVs have also been investigated in animal models as potential vaccine candidates, but none has yet progressed to the stage of clinical trials (13, 28, 29). The active dose applied that induces strong immune response and provides protection is in the wide range of 1 to 500 μg formulated either with or without the addition of adjuvants (30, 31). Different routes of OMVs administration including the intranasal route have been explored (32). More recently, OMVs from different species have been assessed for their adjuvant properties based on the mechanisms of recognition and uptake by antigen-presenting cells, such as dendritic cells and B lymphocytes (33–35). These adjuvant properties can vary between different species due to their non-identical PAMPs composition. In this context, we explored the adjuvant properties of OMVs derived from *B. pertussis* (OMV*_Bp_*) compared to those from other bacterial species, such as those derived from *Escherichia coli* (OMV*_E.coli_*), and a widely used commercial adjuvant in human vaccines known as alum (alhydrogel). Specifically, we evaluated whether OMVs could induce adaptive immunity against co-administered antigens within a single formulation. *In vivo* assays revealed that mice immunized with heterologous antigens mixed with OMVs generated a stronger immune response compared to antigens formulated without an adjuvant. Particularly noteworthy, OMV*_Bp_* induced humoral immune responses that were comparable to those induced by formulations containing alum. Furthermore, unlike alum, OMV*_Bp_* directed the immune response towards a Th1 profile, a distinctive feature from formulations containing OMVs derived from other bacterial species, such as *Escherichia coli*.

## Materials and methods

### Mice

BALB/c mice (4 weeks old), obtained from the Faculty of Veterinary Sciences at the National University of La Plata, were housed in ventilated cages under standardized conditions with controlled daylight, humidity, and temperature. The animals received food and water *ad libitum*. The animal experiments were authorized by the Ethical Committee for Animal Experiments of the Faculty of Science at La Plata National University (approval number 004-06-15, 003-06-15 extended its validity until August 10, 2023 and 2027).

### Isolation and characterization of outer membrane vesicles

OMVs were produced and characterized as previously described (14, 24). Briefly, *B. pertussis* culture samples from the decelerating growth phase were centrifuged and the bacterial pellet obtained was resuspended in 20mM Tris–HCl, 2mM EDTA pH 8.5. The suspension was sonicated (ultrasonic bath) in ice-cold for 20 min. After two centrifugations at 10,000×g for 20 min at 4 °C, the supernatant was pelleted at 100,000×g for 2 h at 4 °C. This pellet was re-suspended in Tris buffer (20 mM pH 7.6). The same protocol was used to obtain OMVs derived from *Escherichia coli*. The samples obtained were negatively stained for electron microscope examination. Protein content was estimated by the Bradford method using bovine serum albumin as standard (36). The presence of the main immunogenic proteins in the OMVs was confirmed by immunoblot assays using specific antibodies as we previously described (19).

### Formulation of OMV-based vaccine

The characterized OMVs, ranging in size from approximately 50 to 200 nanometers, were detoxified with formalin (0.37% at 37°C overnight). To formulate the combined OMV+DTS vaccine, OMVs (at concentrations ranging from 1.5 µg to 6 µg of total OMV protein per dose, as depicted in the figure legends) were formulated with diphtheria (D: 0.45. to 1.8 µg/dose) and tetanus toxoids (T: 2.1 to 8.4 µg/dose), along with the recombinant Spike trimer derived from the ancestral SARS-CoV-2 (S: 0.75 or 2 µg/dose (37, 38). The tetanus and diphtheria toxoids were from the Dr. Tomás Perón Biological Institute, La Plata, Buenos Aires, Argentina, producer of the bacterial double vaccine. The recombinant Spike trimer protein was obtained by our group using the methodologies described previously (37–39). The safety of this vaccine was assessed using a mouse weight-gain test as per WHO 2007 guidelines, as well as murine and human whole-blood IL-6 release assays (25, 40).

For formulations containing aluminum (Alhydrogel, CRODA), the content did not exceed 1.25 mg/dose.

### Immunization of mice, sample collection and tissue harvest

Groups of 6-8 female/male BALB/c mice were immunized with OMVs-based vaccine formulated with heterologous immunogen in 100 µl PBS via intramuscular (i.m.) injection or in 40 µl PBS via the intranasal (i.n.) route using a two-dose schedule.

For the assessment of humoral immune responses, blood was collected from isoflurane-anesthetized mice via the submandibular vein at different time after a vaccination dose. At sacrifice, blood and spleen were collected. Blood was centrifuged at 8,000 g for 10 min to separate serum. Spleens were processed into single-cell suspensions, washed, and resuspended in RPMI medium supplemented with 1% penicillin/streptomycin and 10% FBS to determine cellular response.

### Assessment of humoral immune responses

Levels of anti-immunogen total IgG, IgG isotypes, and IgA in sera were quantified using ELISA. Briefly, 96-well high-binding ELISA plates (Nunc A/S, Roskilde, Denmark) were coated overnight at 4°C with 100 µL of 0.75 µg/mL Diphtheria toxin, 0.5 µg/mL Tetanus toxin or 0.45 µg/mL Spike protein. Plates were washed five times with PBS/0.05% Tween20 (PBS-T), and then blocked for 1 h at 37°C with 200 µL 3% skimmed milk powder in PBS before incubation with different diluted samples of mouse serum (1 h, 37°C). To measure IgA, IgG, and IgG isotypes after five washes with PBS-T (Sigma-Aldrich), the bound antibody was incubated with 100 µL of horseradish-peroxidase labeled anti-mouse IgG at 1:8,000, subclass-specific anti-mouse IgA at 1/750, IgG1 at 1:3,000, or anti-mouse IgG2a at 1:750 (Sigma, Aldrich). After five washes with PBS-T, 100 µL/well of the substrate o-phenylenediamine dihydrochloride (OPD, Sigma-Aldrich) diluted in 0.05 M citrate buffer (pH 5.0) was added. Plates were developed for 15 min at RT in the dark. The reaction was stopped with 50 µL/well of 4 N H_2_SO_4_. Bound IgG were detected spectrophotometrically at 490 nm (Titertek Multiskan Model 340 microplate reader ICN, USA).

### Assessment of cellular immune responses

The cellular response was analyzed as previously described (25). Briefly, spleen cells from immunized and non-immunized mice were harvested 4 weeks after the last immunization and seeded in 48-well culture plates at 10^6^ per well in a volume of 500 μL of RPMI 1640 cell-culture medium supplemented with 10% (v/v) fetal-bovine serum (Invitrogen, Buenos Aires Argentina) containing 100 IU/mL penicillin and 100μg/mL streptomycin. All the spleen cells were either stimulated with the different heterologous immunogen (2 µg/well for D and T and 1 µg/well for S), or medium only. Supernatants were removed after 72 h of incubation at 37°C in an atmosphere of 5% (v/v) CO2/air and the production of IFNγ, and IL-5 determined by ELISA (Mabtech, USA), according to the conditions specified by the manufacturer.

### Statistical analysis

All statistics were analyzed using GraphPad Prism 9. Ordinary one- or two-way ANOVA was performed to determine statistical significance between groups of three or more (Tukey’s multiple comparison test or Bonferroni´s multiple comparison test as appropriate). Statistical differences between groups of two were determined via unpaired t test for data deemed normal. Differences were significant when p< 0.05.

## Results

### Adjuvant properties of OMV*_Bp_*

**Figure 1:**
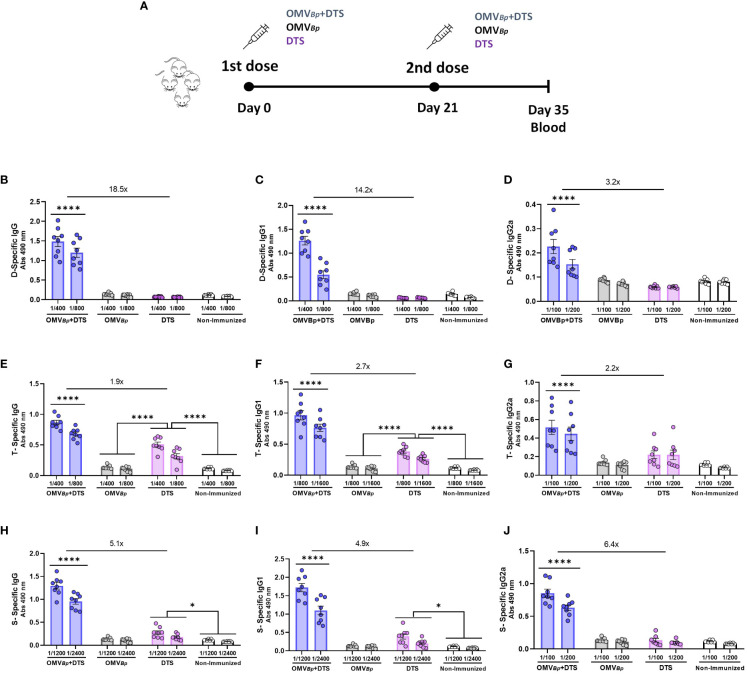
Specific humoral immune response in mice immunized with formulations containing OMV*_Bp_* as adjuvant. Schematic representation of vaccination schedule is presented in **(A)**. Female BALB/c mice (n=8) were intramuscularly vaccinated with 2 doses of D (0.45 μg/dose) T (2.1 μg/dose) S (0.75 μg/dose) formulated with or without OMV*_Bp_* (3 μg/dose) at days 0 and 21. OMV*_Bp_* without any heterologous immunogen immunized mice and non-immunized mice were used as controls. The levels of D-specific IgG **(B)**, IgG1 **(C)**, and IgG2a **(D)**, T-specific IgG **(E)**, IgG1 **(F)**, and IgG2a **(G)** and S-specific IgG **(H)**, IgG1 **(I)**, and IgG2a **(J)** induced by the two dose schedules here tested were analyzed by ELISA in sera collected on Day 14 after the last dose (absorbance values at 490 nm for 2 sera dilutions). The increases detected in IgG levels and isotypes for formulations containing OMV*_Bp_* compared to formulations containing DTS alone are indicated at the top of the figures. ****p<0.0001, *p<0.05 by two way ANOVA using Bonferroni for multiple comparisons.

To assess the adjuvant properties of the OMVs derived from *B. pertussis* (OMV*_Bp_*) we conducted *in vivo* assays using a murine immunization model with formulations containing OMVs and heterologous immunogens. Specifically, we evaluated the immune response induced by formulations containing 3 heterologous immunogens: Diphtheria toxoid (D), Tetanus toxoid (T), and the fully glycosylated Spike protein of SARS-CoV-2 (S). Two doses of the tested formulations were administered intramuscularly (final volume 100 µl), spaced 21 days apart (**Figure 1A**). Control groups of mice were immunized as follows: one subgroup received a formulation containing solely the heterologous immunogens (DTS), another subgroup received OMV*_Bp_* alone without any immunogen, while a separate group received no immunological treatment.

At 14 days after the second immunization, we evaluated the specific IgG, IgG1 (a marker for Th2 response), and IgG2a (a marker for Th1 response) levels for each of the heterologous immunogens tested in this study. The specific humoral immune response to Diphtheria toxoid is depicted in **Figures 1B–D**. While IgG levels were either undetectable in non-immunized and control OMV*_Bp_*-treated mice groups or very low in DTS-treated mice, significantly high D-specific IgG levels were observed in OMV*_Bp_* +DTS immunized group (**Figure 1B**). The highest levels of D-specific IgG1 (**Figure 1C**) and IgG2a (**Figure 1D**) were also detected in the OMV*_Bp_* +DTS treated group. Conversely, the lowest levels of D-specific IgG1 and IgG2a were detected for the 2-dose DTS regimen. In both the non-immunized mice and the control groups treated with OMV*_Bp_* alone, the levels of IgG isotypes were undetectable. Similar results were obtained when analyzing the specific IgG, IgG1, and IgG2a responses for T (**Figures 1E–G**) and for S (**Figures 1H–J**). The highest levels of T- or S-specific IgG (**Figures 1E, H**, respectively), IgG1 (**Figures 1F, I**, respectively), and IgG2a (**Figures 1G, J**, respectively) were also detected in the OMVBp+DTS-treated group.

**Figure 2:**
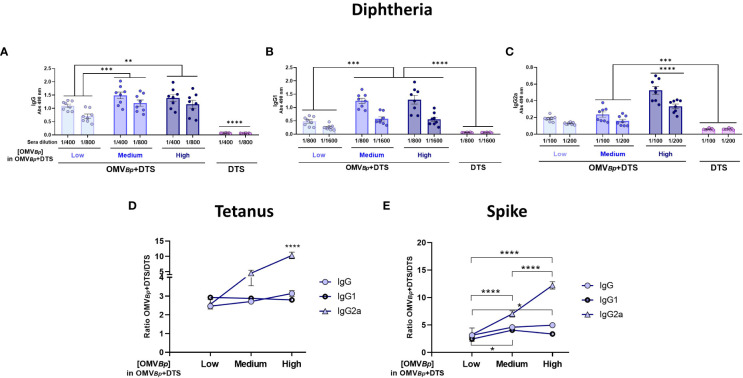
Specific humoral immune response in mice immunized with formulations containing different amounts of OMV*_Bp_* as adjuvant. Mice were immunized with 2 doses of D (0.45 μg/dose) T (2.1 μg/dose) S (0.75 μg/dose) formulated with high (6 μg of protein per dose), medium (3 μg of protein per dose) or low (1.5 μg of protein per dose) quantities of OMV*_Bp_*, or DTS alone as control. Sera were collected 14 days after the last dose. The levels of Diphtheria-specific IgG, IgG1, and IgG2a are presented in **(A–C)**, respectively (absorbance values at 490 nm for 2 sera dilutions). The specific humoral immune response against Tetanus toxoid **(D)** and Spike protein **(E)** are shown as the specific fold increase obtained for each formulation containing OMV*_Bp_* in relation DTS formulation alone. ****p<0.0001, ***p<0.001, **p<0.01 *p<0.05 by two way ANOVA using Bonferroni for multiple comparisons.

To further delineate the adjuvant effect of OMV*_Bp_*, we conducted a dose-response assay using different OMV*_Bp_* concentrations: low (1.5 μg of protein per dose), medium (3 μg per dose), and high (6 μg per dose) (**Figure 2**). In this assay, although a dose-response effect was observed, the D-specific IgG levels in animals immunized with OMV*_Bp_* +DTS were significantly higher than those detected in animals immunized with DTS alone, irrespective of the OMV*_Bp_* concentration used in the formulation (**Figure 2A**). Regarding IgG1 (**Figure 2B**) and IgG2a (**Figure 2C**), levels detected in animals immunized with formulations containing medium and high concentrations of OMV*_Bp_* were significantly higher than those in animals receiving the lowest OMV*_Bp_* concentration and those treated only with DTS (p<0.001). In the non-treated or OMV*_Bp_* control groups, IgG, IgG1, and IgG2a were undetectable (not shown). These controls consistently showed undetectable levels throughout the study and will not be further mentioned to prevent redundancy.

These analyses were extended to Tetanus (**Figure 2D**) and Spike protein (**Figure 2E**) to assess the breadth of OMV*_Bp_* adjuvant properties and determine their most suitable OMV*_Bp_* concentration to demonstrate this effect. For both heterologous immunogens, the OMV*_Bp_* +DTS formulation induced at least a 2-fold increase of IgG, IgG1 and IgG2a in comparison with DTS formulation (p<0.001). In the case of IgG2a, a dose-response effect of the OMV*_Bp_* was observed, with the highest levels detected for the highest OMV*_Bp_* concentration (**Figures 2D, E**). The data from the individual determinations of IgG, IgG1, and IgG2a for T and S are presented in the **Supplementary Material** (**Supplementary Figures S1A–F**, respectively).

**Figure 3:**
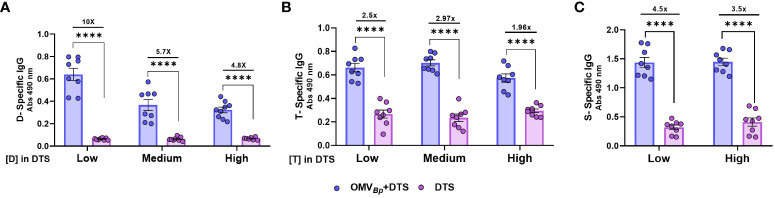
Specific humoral immune response in mice immunized with formulations containing different amounts of the immunogens combined with OMV*_Bp_* as adjuvant. Mice were immunized with 2 doses of formulations containing low (D: 0.45 μg/dose. T: 2.1 μg/dose S: 0.75 μg/dose), medium (double for D and T the low) or high (quadruple the low for D and T and 2 μg/dose for S) quantities of DTS immunogens formulated with or without OMV*_Bp_*. Sera were collected 14 days after the last dose. The levels of D-specific IgG **(A)**, T-specific IgG **(B)**, S-specific IgG **(C)** are presented as absorbance values at 490 nm. The increases detected in specific IgG levels and isotypes for formulations containing OMV*_Bp_*, compared to formulations containing DTS alone, are indicated at the top of the figures for each of the tested immunogen concentrations. ****p<0.0001 correspond to comparison of each concentration of DTS with and without OMV*_Bp_* by two way ANOVA using Bonferroni for multiple comparisons.

Considering that adequate levels of IgG are achieved with the medium concentration of OMV*_Bp_* (3 μg of protein per dose) and that doubling the OMV*_Bp_* concentration does not lead to a twofold increase, we decided to use the medium concentration of OMV*_Bp_* to conduct a dose-response assay for the immunogens. The minimum concentration tested for each immunogen corresponds to 0.45 μg/dose for D, 2.1 μg/dose for T, and 0.75 μg/dose for S. These quantities were previously reported as immunogenic (39, 41, 42). The medium concentration tested here is twice that amount for T and D, and the high concentration is four times the minimum tested for T and D and 2 μg/dose for S. For these experiments, we once again employed a schedule of 2 doses spaced 21 days apart, and at 14 days after the last dose, blood was collected to analyze specific IgG levels for each immunogen in the serum. As shown in **Figure 3**, the highest increase in specific IgG levels for the OMV*_Bp_* +DTS formulation was observed when the lowest levels of D and S were used. For Diphtheria, an increase of at least 4.8 times was detected (**Figure 3A**), and for Spike, it was 3.5 times (**Figure 3C**). In the case of Tetanus, an increase in IgG levels of at least 1.9 times was observed in the presence of OMVBp (**Figure 3B**).

All these results demonstrate the broad adjuvant properties of OMV*_Bp_*, leading to a mixed Th1 (IgG2a)/Th2 (IgG1) immune response profile.

### Comparative analysis of the adjuvant properties of OMV*_Bp_* with OMVs from *E. coli* and alum

**Figure 4:**
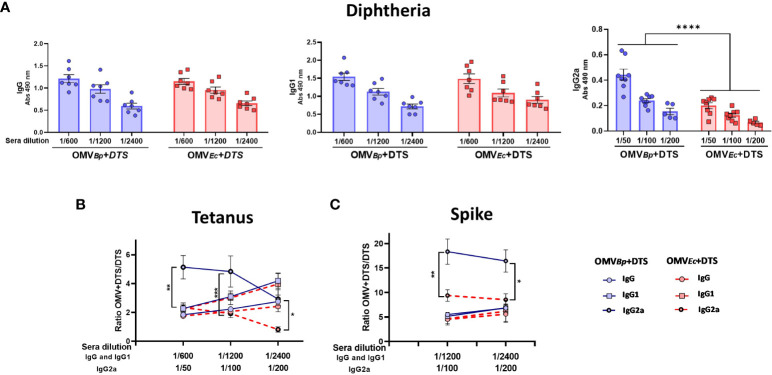
Comparison of adjuvant properties between OMVs derived from *B*. *pertussis* and those from *E*. *coli*. The levels of D-specific IgG, IgG1, and IgG2a induced after the second dose of formulations containing OMVs (3 μg) from different sources as adjuvants are presented in **(A)** The quantities of the heterologous immunogens used in the formulations were the minimum amounts tested in our study (D: 0.45 μg/dose. T: 2.1 μg/dose S: 0.75 μg/dose). The levels of the different immunoglobulins were determined in sera collected 14 days after the last dose by ELISA. The comparison between the levels of specific IgG and isotypes for formulations containing OMVs and those for formulations containing DTS alone is presented in **(B)** for Tetanus toxoid and **(C)** for Spike protein. Continuous blue lines represent the specific fold increase obtained for formulations containing OMV*_Bp_* compared to the DTS formulation alone. Discontinuous red lines represent the specific fold increase obtained for formulations containing OMV*_E.coli_* compared to the DTS formulation alone. ****p<0.0001, ***p<0.001, **p<0.01, *p<0.05 by two-way ANOVA using Bonferroni for multiple comparisons.

With the aim of analyzing whether the observed adjuvant properties of *B. pertussis* OMVs extend to OMVs derived from another bacterial species, we conducted the aforementioned *in vivo* assays using formulations containing OMVs derived from *E. coli* (OMV*_E.coli_* +DTS). **Figure 4A** presents the comparative data between the levels of D-specific IgG, IgG1, and IgG2a isotypes induced by the formulation containing the OMV*_Bp_* and those corresponding to the treatment containing the OMV*_E.coli_*. The D- specific IgG and IgG1 levels were high and similar among the groups treated with formulations containing the different OMVs. It was interesting to note that for the group of animals treated with formulations containing OMV*_Bp_*, the D- specific IgG2a levels were significantly higher (p<0.0001) than those detected for the group treated with OMV*_E.coli_* +DTS, indicating that OMV*_Bp_* triggers the immune response towards a Th1 profile (**Figure 4A**).

Similar results were obtained when IgG and the respective isotypes were measured for T (**Figure 4B**) and S (**Figure 4C**). While no differences in the ratio of IgG and IgG1 levels for both immunogens were detected between formulations containing either OMV*_Bp_* or OMV*_E.coli_* compared to DTS alone, a higher fold increase in IgG2a was observed for OMV*_Bp_* compared to that detected for mice immunized with OMV*_E.coli_* +DTS (p<0.05). The data from the individual determinations of IgG, IgG1, and IgG2a for T and S are presented in the **Supplementary Material** (**Supplementary Figures S2A, B**, respectively).

**Figure 5:**
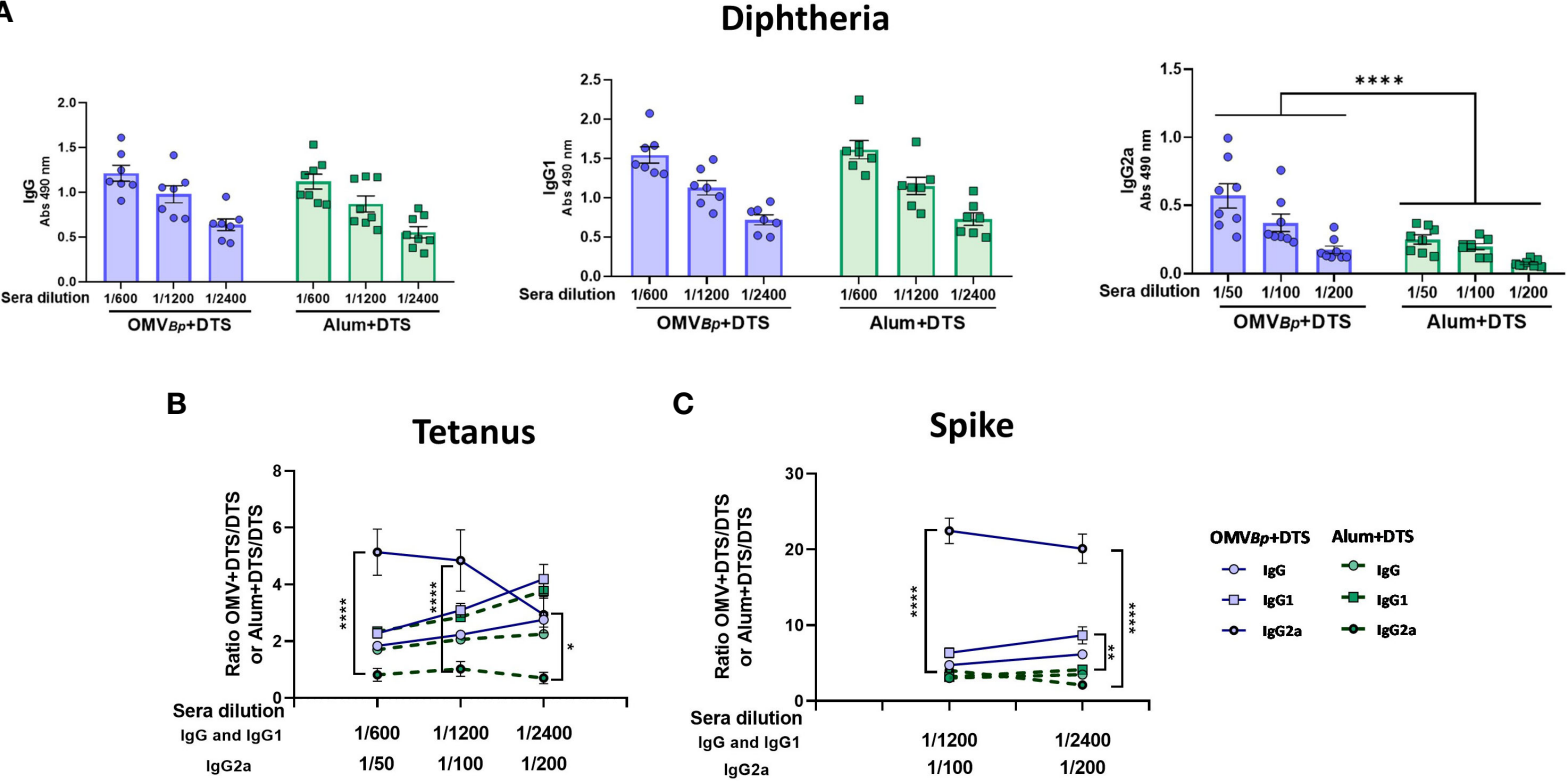
Comparison of adjuvant properties between OMVs derived from *B*. *pertussis* and Alum. The levels of D-specific IgG, IgG1, and IgG2a induced after the second dose of formulations containing OMV*_Bp_* or Alum (alhydrogel) as adjuvants are presented in **(A)**. For these assays the quantities of the heterologous immunogens used in the formulations were the minimum amounts tested in our study (D: 0.45 μg/dose. T: 2.1 μg/dose S: 0.75 μg/dose). The levels of the different immunoglobulins were determined in sera collected 14 days after the last dose by ELISA. The comparison between the levels of specific IgG and isotypes for formulations containing OMV*_Bp_* or Alum and those for formulations containing DTS alone is presented in **(B)** for Tetanus toxoid and **(C)** for Spike protein. Continuous blue lines represent the specific fold increase obtained for formulations containing OMV*_Bp_* compared to the DTS formulation alone. Discontinuous green lines represent the specific fold increase obtained for formulations containing Alum compared to the DTS formulation alone. ****p<0.0001, **p<0.01, *p<0.05 by two-way ANOVA using Bonferroni for multiple comparisons.

Comparative assays were also conducted between formulations containing OMV*_Bp_* and a well-known adjuvant, alum (Alhydrogel) (**Figure 5**). Similar levels of specific IgG and IgG1 against D were observed for both OMV*_Bp_* and alum adjuvants (**Figure 5A**). However, notably, the OMV*_Bp_*-based vaccine exhibited a higher increase in specific IgG2a compared to the alum formulation (p<0.0001).

**Figure 6:**
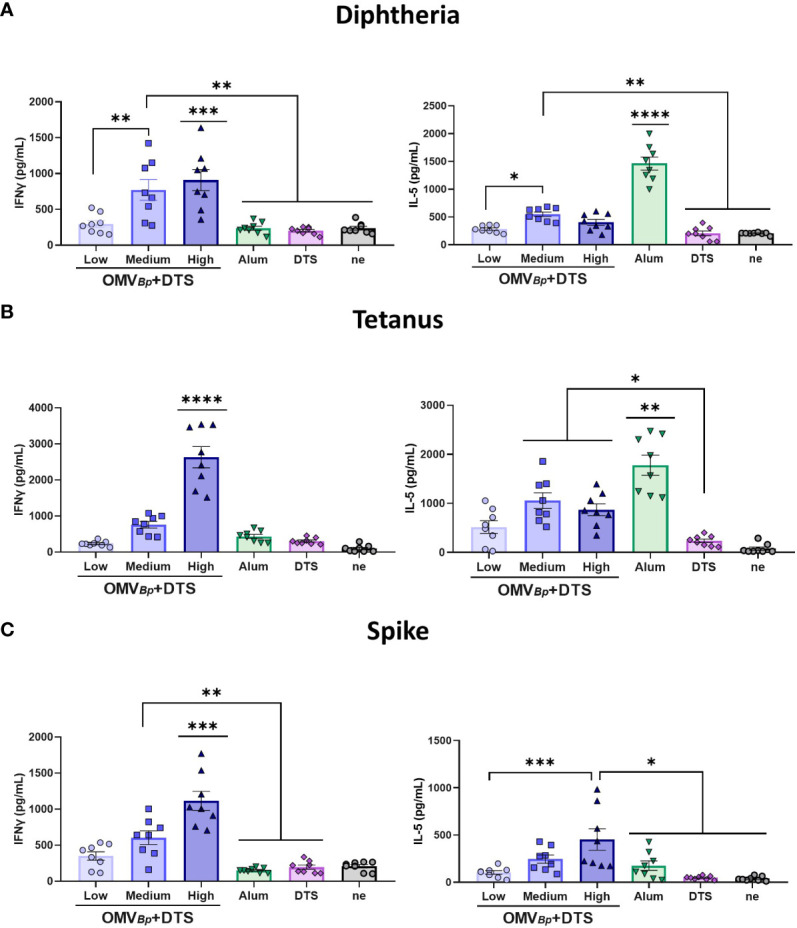
OMV*_Bp_* formulation induces Ag-specific Th1 (IFN-γ) while alum formulation mainly induces Th2 (IL-5). BALB/c mice (n=8/group) were immunized on Days 0 and 21 with formulations containing different quantities of OMV*_Bp_* (high: 6 μg/dose, medium: 3 μg/dose or low: 1.5 μg/dose) and the lower DTS quantities here assayed (D: 0.45 μg/dose. T: 2.1 μg/dose S: 0.75 μg/dose). Levels of secreted IFN-γ and IL-5 following splenocytes stimulation with medium or D **(A)**, T **(B)** or S **(C)** were determined by ELISA. Bars are means ± SEM of pg/ml. **** p<0.0001, *** p<0.001, **p<0.01, *p<0.05 by two-way ANOVA using Bonferroni for multiple comparisons.

In **Figures 5B, C**, IgG, IgG1, and IgG2a against T and S (expressed as ratio of adjuvanted to antigen-alone formulations) are presented. The data from the individual determinations are presented in the **Supplementary Material** (**Supplementary Figures S3A, B**, respectively). Once again, it was observed that the formulation based on OMV*_Bp_* differs from that containing alum in directing the response mainly towards Th1, as it exhibits higher levels of IgG2a. For these formulations, the levels of IFNγ and IL-5 were also evaluated in spleen cell stimulation assays (**Figure 6**). Consistent with the humoral immune response, formulations containing the OMV*_Bp_* exhibited for D (**Figure 6A**) and for T (**Figure 6B**) and S (**Figure 6C**) the highest IFNγ levels, while the alum-containing formulation induced significantly higher IL-5 levels (p<0.05).

### Adjuvant properties of OMV*_Bp_* administered via the intranasal route

**Figure 7:**
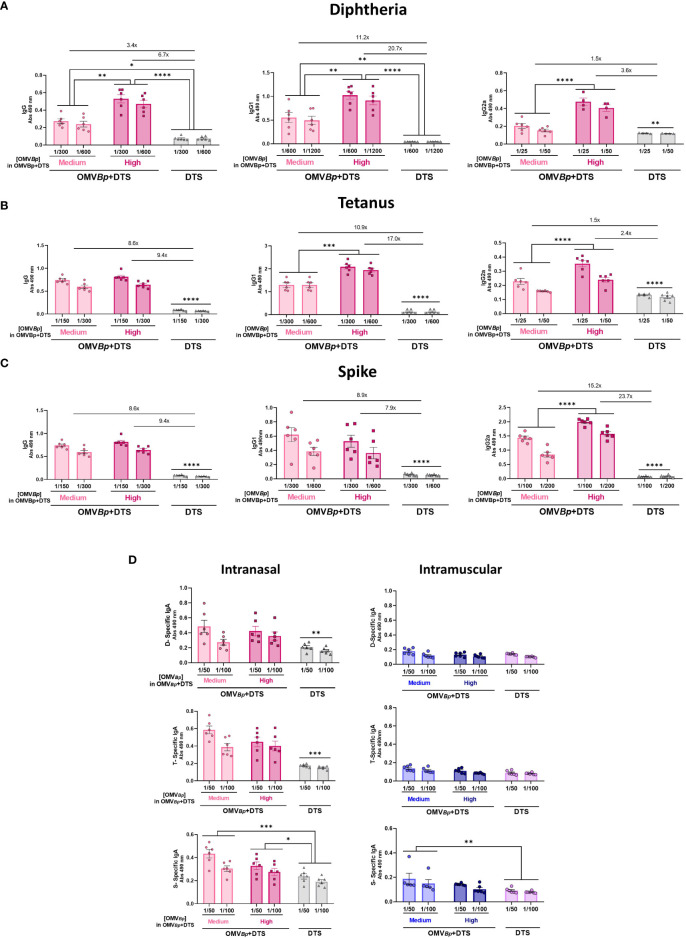
Specific humoral immune response in mice immunized by intranasal route with formulations containing OMV*_Bp_* as adjuvant. Female BALB/c mice (n=6) were intranasally vaccinated with 2 doses of DTS (at the minimum quantity tested in our study) formulated with or without OMV*_Bp_* (3 or 6 μg/dose) at days 0 and 21. The levels of D-specific IgG, IgG1 and IgG2a **(A)**, T-specific IgG, IgG1 and IgG2a **(B)**, S-specific IgG, IgG1 and IgG2a **(C)** induced by the two dose schedules here tested were analyzed by ELISA in sera collected on Day 14 after the last dose (absorbance values at 490 nm for 2 sera dilutions). The increases detected in IgG levels and isotypes for formulations containing OMV*_Bp_* compared to formulations containing DTS alone are indicated at the top of the figures. The levels of D-specific IgA, T-specific IgA, and S-specific IgA induced by the two dose schedules via the intranasal or intramuscular route are also presented in panel **(D)**. ****p<0.0001, ***p<0.001, **p<0.01, *p<0.05 by two-way ANOVA using Bonferroni for multiple comparisons.

Finally, we assessed the adjuvant properties of OMV*_Bp_* administered via mucosal route (**Figure 7**). To achieve this, we employed a murine model with a two-dose immunization scheme, where the formulations to be tested were administered intranasally. Specifically, we compared the levels of specific IgG induced by OMV*_Bp_* +DTS (at medium and high concentrations of OMV*_Bp_*) for the three tested immunogens with those induced by the formulation containing only DTS. For both formulations, the DTS concentration corresponds to the lowest concentration used in this study. The specific IgG levels for the three immunogens induced by formulations containing both medium and high OMV*_Bp_* concentrations were significantly higher than those induced by DTS (p<0.05) (**Figure 7**). Specifically, for the high dose of OMV*_Bp_*, the specific IgG increments compared to those found for DTS were at least: 3 times for D (**Figure 7A**), 8 times for T (**Figure 7B**), and 18 times for S (**Figure 7C**). For all heterologous immunogens tested, OMV*_Bp_* +DTS intranasal immunization also resulted in significantly higher levels of IgG1 and IgG2a compared to those detected in animals immunized with the immunogens alone (**Figure 7**). Furthermore, in these animals treated with formulations containing OMV*_Bp_*, specific IgA levels were higher than those detected in animals treated with DTS alone (p<0.05) (**Figure 7D**). IgA levels measured for comparative purposes in intramuscularly immunized animals were practically undetectable for all treatments (**Figure 7D**).

## Discussion

Since the first evidence of adjuvants in 1926, when Alexander Glenny found that mixing aluminum salts with antigens and injecting them into guinea pigs induced more antibodies than administering antigens alone, the development of adjuvants was limited (43). It wasn’t until 1997 that the oil-in-water emulsion MF59 was licensed in Europe as an adjuvant for influenza vaccines. Over the next 20 years, four additional adjuvants (AS04, AS03, AS01, and CpG ODN 1018) were licensed for use in vaccines, expanding the variety of adjuvants available for human vaccines (44). Additionally, many other different classes of compounds have been evaluated as adjuvants during this time, including microbial products, emulsions, saponins, synthetic small molecule agonists, polymers, nanoparticles, and among them OMVs and liposomes (45–48). The adjuvant properties of OMVs were highlighted in the development of vaccines against infections caused by *N. meningitidis* serogroup B (49). For this serogroup, using the capsular polysaccharide coupled to a carrier protein is not feasible due to the risk of autoimmunity (4, 50, 51). Instead, a formulation incorporating the PorA protein with OMV as an adjuvant was successfully developed (8, 52).. Based on these findings, as well as on OMV properties such as the activation and maturation of professional antigen-presenting cells, the activation of the inflammasome pathway, and their size and composition, which includes various PAMPs, it has been generally proposed that OMVs have an adjuvant role (2, 35, 53, 54). However, the potency of this property and the characterization of the immune response are expected to vary depending on the source of these OMVs.

Here, we have demonstrated that OMV*_Bp_*, possessing its own lipooligosaccharide (LOS, one of the OMVs PAMPs), is a potent inducer of immune responses against co-delivered heterologous immunogens, including Tetanus and Diphtheria toxoids, as well as the SARS-CoV-2 Spike protein. Regardless of the specific immunogen under scrutiny, the experiments consistently revealed that OMV*_Bp_* triggered a significant increase in IgG levels compared to animals immunized solely with the respective heterologous immunogens. These heightened IgG levels were comparable to those induced by formulations containing alum, a well-established adjuvant widely used in human vaccine formulations (55). The potency of OMV*_Bp_* as an adjuvant was also evidenced by the fact that doses as low as 1.5 μg of protein per dose caused a significant increase in induced adaptive immunity against the different co-administered immunogens here tested. The dose-response assays for the heterologous immunogens revealed that at lower doses of these antigens, the adjuvant properties of the OMVs were either higher or comparable to that observed when testing intermediate or high doses of the immunogens. These results underscore the value of OMVs as they may contribute to antigen sparing, a critical aspect in epidemic or pandemic scenarios of infectious diseases. In these scenarios, to protect a naive global population against pandemic diseases, vaccines should be effective at low antigen doses due to limited manufacturing capacity.

Another significant finding was that combining OMV*_Bp_* with an unrelated protein resulted in an enhanced IgG2a response specific to the co-administered antigen, surpassing that observed in formulations containing the immunogens alone. This increase in IgG2a was notably amplified at the medium (3 μg) and high (6 μg). OMV*_Bp_* concentrations tested in this study. This bias toward a Th1 profile was further evidenced by the levels of IFNγ detected in spleen cell stimulation assays, which not only differed from those detected for alum but also when using another OMV derived from a different bacterial species (OMV*_E.coli_*). For both OMV formulations, the adjuvant role for various co-administered immunogens was demonstrated, although the bias toward a Th1 profile was less potent for OMV*_E.coli_*. This discrepancy may be attributed to the diversity of PAMPs composition between the two OMVs. Specifically, OMVBp contains molecules such as lipooligosaccharides (LOS) and lipoprotein BP1569 that have been described as immunomodulators, shaping the response towards a Th1 profile (56, 57). In fact, these components have been proposed as vaccine components to induce the Th1 profile (56, 58), thereby overcoming the limitation of current acellular vaccines that mostly induce a Th2 profile (57). The skewed Th1 response is a highly significant result because achieving this response without the use of a live or replicating vaccine is challenging. Importantly, this response was achieved without directly linking or conjugating the antigens to the OMVs. In fact recent observations indicate that the inclusion of an heterologous immunogen to the OMV composition (recombinant *E. coli*-derived OMVs) or the use of formulations comprising OMVs plus soluble heterologous immunogen (*E. coli* OMVs + antigen mixtures) does not seem to result in discernible differences in immunogenicity (30).

Our data show that the addition of OMV*_Bp_* to vaccine formulation could potentially enhance humoral and cellular immune responses to a variety of immunogens derived from different pathogens. Moreover, we observed that OMV*_Bp_* exhibited adjuvanticity through multiple routes, including intramuscular injection, commonly used in traditional vaccines, as well as the intranasal route.

The incorporation of OMV adjuvants into new or existing vaccines shows promise in enhancing both the magnitude and breadth of adaptive immune responses, thereby elevating overall vaccine efficacy. Regarding OMV*_Bp_*, its dual role as both a vaccine antigen and adjuvant is noteworthy, potentially opening doors for its inclusion in combined formulations. Such potential could reduce costs and streamline vaccine distribution and administration strategies within the population.

## Data availability statement

The original contributions presented in the study are included in the article/**Supplementary Material**. Further inquiries can be directed to the corresponding author.

## Ethics statement

The animal study was approved by Ethical Committee for Animal Experiments of the Faculty of Science at La Plata National University (approval number 004-06-15, 003-06-15 extended its validity until August 10, 2023 and 2027). The study was conducted in accordance with the local legislation and institutional requirements.

## Author contributions

BP: Investigation, Methodology, Writing – review & editing. LL: Methodology, Writing – review & editing. OL: Methodology, Writing – review & editing. PA: Methodology, Writing – review & editing. EZ: Investigation, Methodology, Writing – review & editing. MS: Methodology, Writing – review & editing. YD: Methodology, Writing – review & editing. DH: Conceptualization, Formal analysis, Funding acquisition, Investigation, Project administration, Supervision, Writing – original draft, Writing – review & editing.

## Supplementary material

Supplementary Figure 1 Specific humoral immune response in mice immunized with formulations containing different amounts of OMV*_Bp_* as adjuvant. Mice were immunized with 2 doses of D (0.45 μg/dose) T (2.1 μg/dose) S (0.75 μg/dose) formulated with high (6 μg of protein per dose), medium (3 μg of protein per dose) or low (1.5 μg of protein per dose) quantities of OMV*_Bp_*, or DTS alone as control. Sera were collected 14 days after the last dose. The levels of Tetanus-specific IgG, IgG1, and IgG2a are presented in **(A–C)**, respectively (absorbance values at 490 nm) and those specific for S in panels **(D, E)** and **(F)**. ****p<0.0001, ***p<0.001, **p<0.01 by two way ANOVA using Bonferroni for multiple comparisons.

Supplementary Figure 2 Comparison of adjuvant properties between OMVs derived from *B. pertussis* and those from *E. coli*. The levels of T-specific or S-specific IgG, IgG1, and IgG2a induced after the second dose of formulations containing OMVs (3 μg) from different sources as adjuvants are presented in **(A, B)**, respectively. The quantities of the heterologous immunogens used in the formulations were the minimum amounts tested in our study (D: 0.45 μg/dose. T: 2.1 μg/dose S: 0.75 μg/dose). The levels of the different immunoglobulins were determined in sera collected 14 days after the last dose by ELISA. ***p<0.001 **p<0.01, by two-way ANOVA using Bonferroni for multiple.

Supplementary Figure 3 Comparison of adjuvant properties between OMVs derived from *B. pertussis* and Alum. The levels of T-specific or S-specific IgG, IgG1, and IgG2a induced after the second dose of formulations containing OMV*_Bp_* or Alum as adjuvants are presented in **(A, B)**, respectively. For these assays the quantities of the heterologous immunogens used in the formulations were the minimum amounts tested in our study (D: 0.45 μg/dose. T: 2.1 μg/dose S: 0.75 μg/dose). The levels of the different immunoglobulins were determined in sera collected 14 days after the last dose by ELISA. ****p<0.0001, ***p<0.001, by two-way ANOVA using Bonferroni for multiple comparisons.
