# Supplementary figures and images for: Outer membrane vesicles derived from Bordetella pertussis are potent adjuvant that drive Th1-biased response
Source: Front Immunol. 2024 Apr 8;15:1387534. doi: 10.3389/fimmu.2024.1387534 (PMC11033331; doi:10.3389/fimmu.2024.1387534)

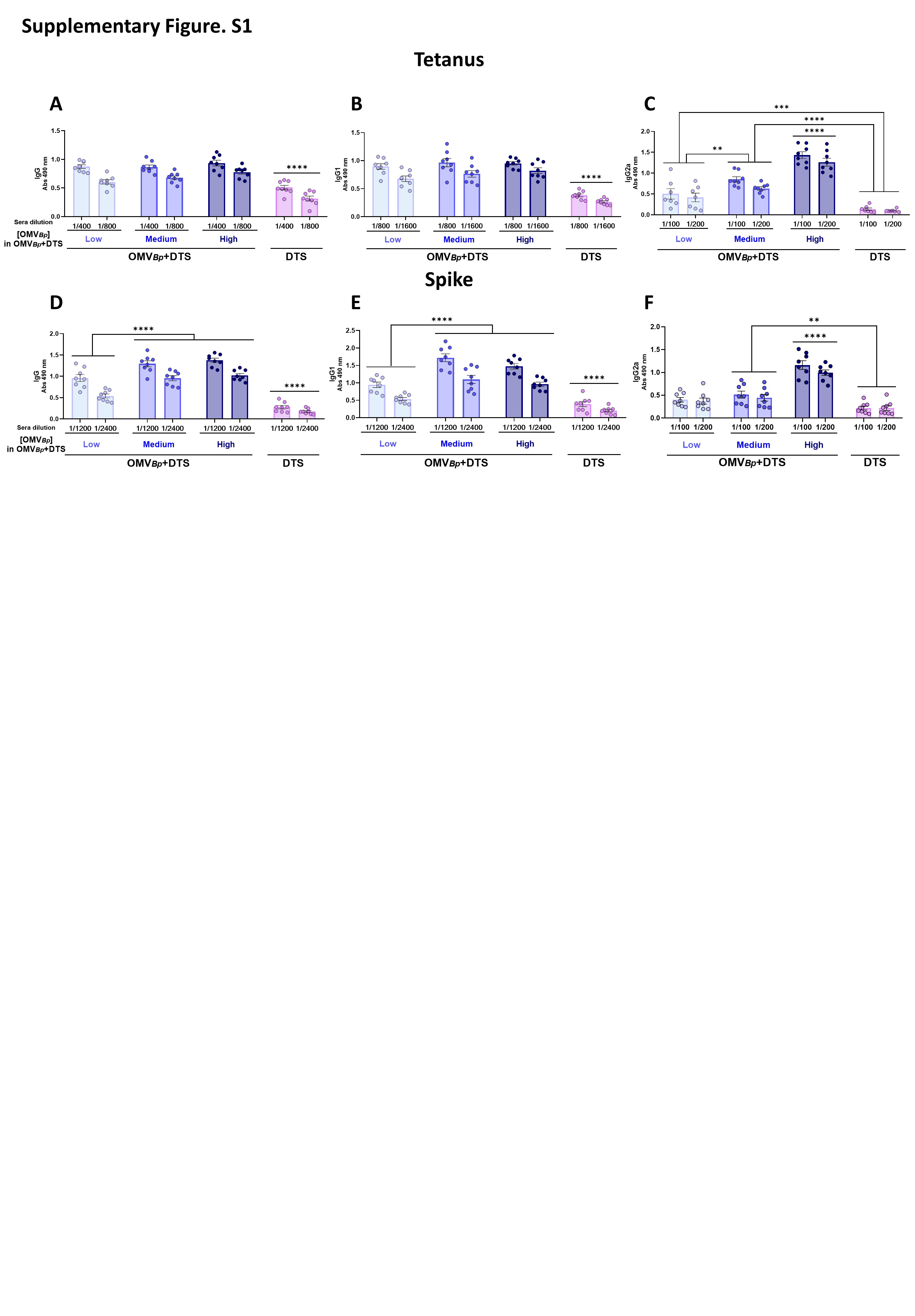

Supplement: Supplementary Figure 1 — Specific humoral immune response in mice immunized with formulations containing different amounts of OMV Bp as adjuvant. Mice were immunized with 2 doses of D (0.45 μg/dose) T (2.1 μg/dose) S (0.75 μg/dose) formulated with high (6 μg of protein per dose), medium (3 μg of protein per dose) or low (1.5 μg of protein per dose) quantities of OMV Bp , or DTS alone as control. Sera were collected 14 days after the last dose. The levels of Tetanus-specific IgG, IgG1, and IgG2a are presented in (A–C), respectively (absorbance values at 490 nm) and those specific for S in panels (D, E) and (F). ****p<0.0001, ***p<0.001, **p<0.01 by two way ANOVA using Bonferroni for multiple comparisons. [file Image_1.jpeg]

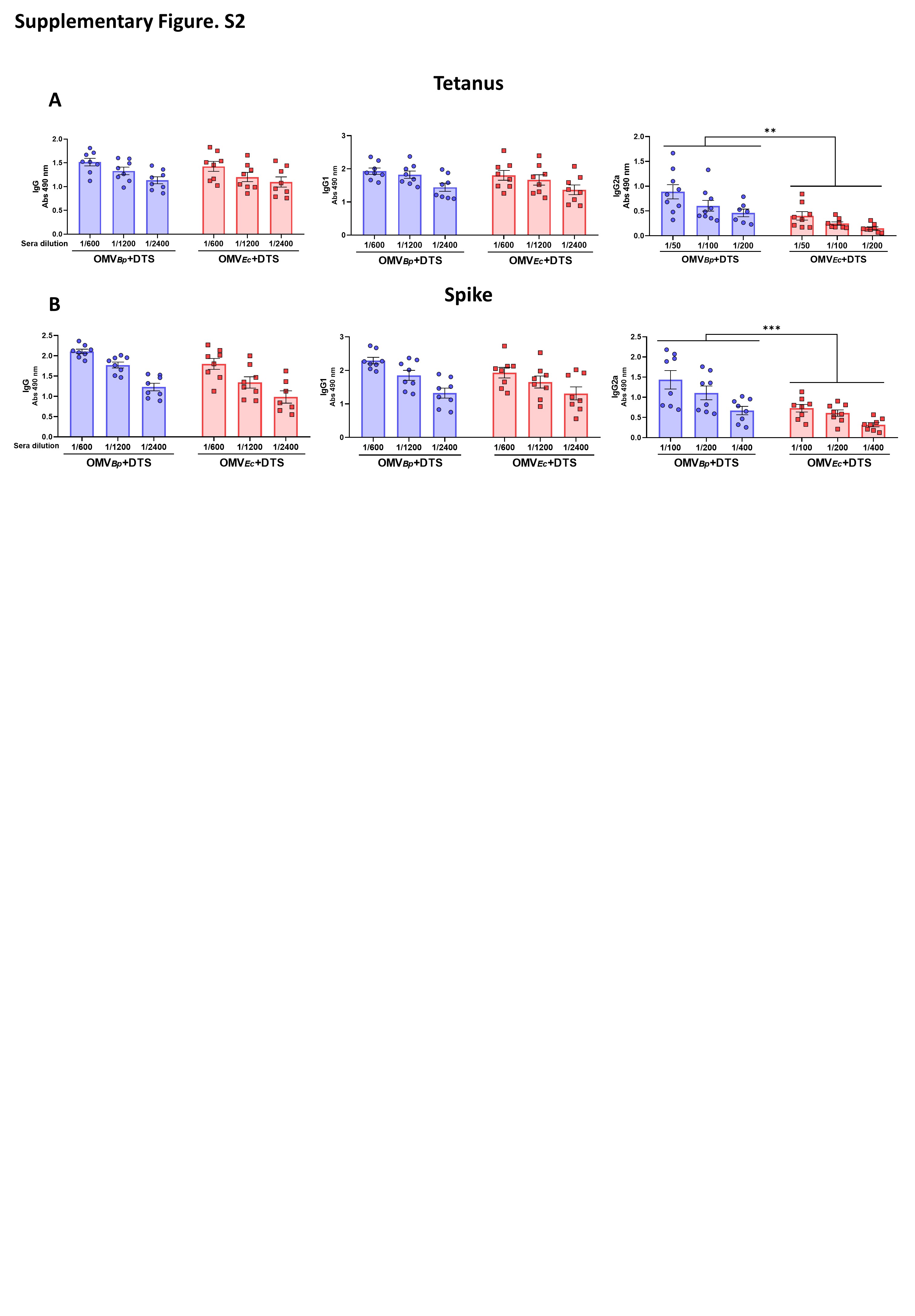

Supplement: Supplementary Figure 2 — Comparison of adjuvant properties between OMVs derived from B. pertussis and those from E. coli. The levels of T-specific or S-specific IgG, IgG1, and IgG2a induced after the second dose of formulations containing OMVs (3 μg) from different sources as adjuvants are presented in (A, B), respectively. The quantities of the heterologous immunogens used in the formulations were the minimum amounts tested in our study (D: 0.45 μg/dose. T: 2.1 μg/dose S: 0.75 μg/dose). The levels of the different immunoglobulins were determined in sera collected 14 days after the last dose by ELISA. ***p<0.001 **p<0.01, by two-way ANOVA using Bonferroni for multiple. [file Image_2.jpeg]

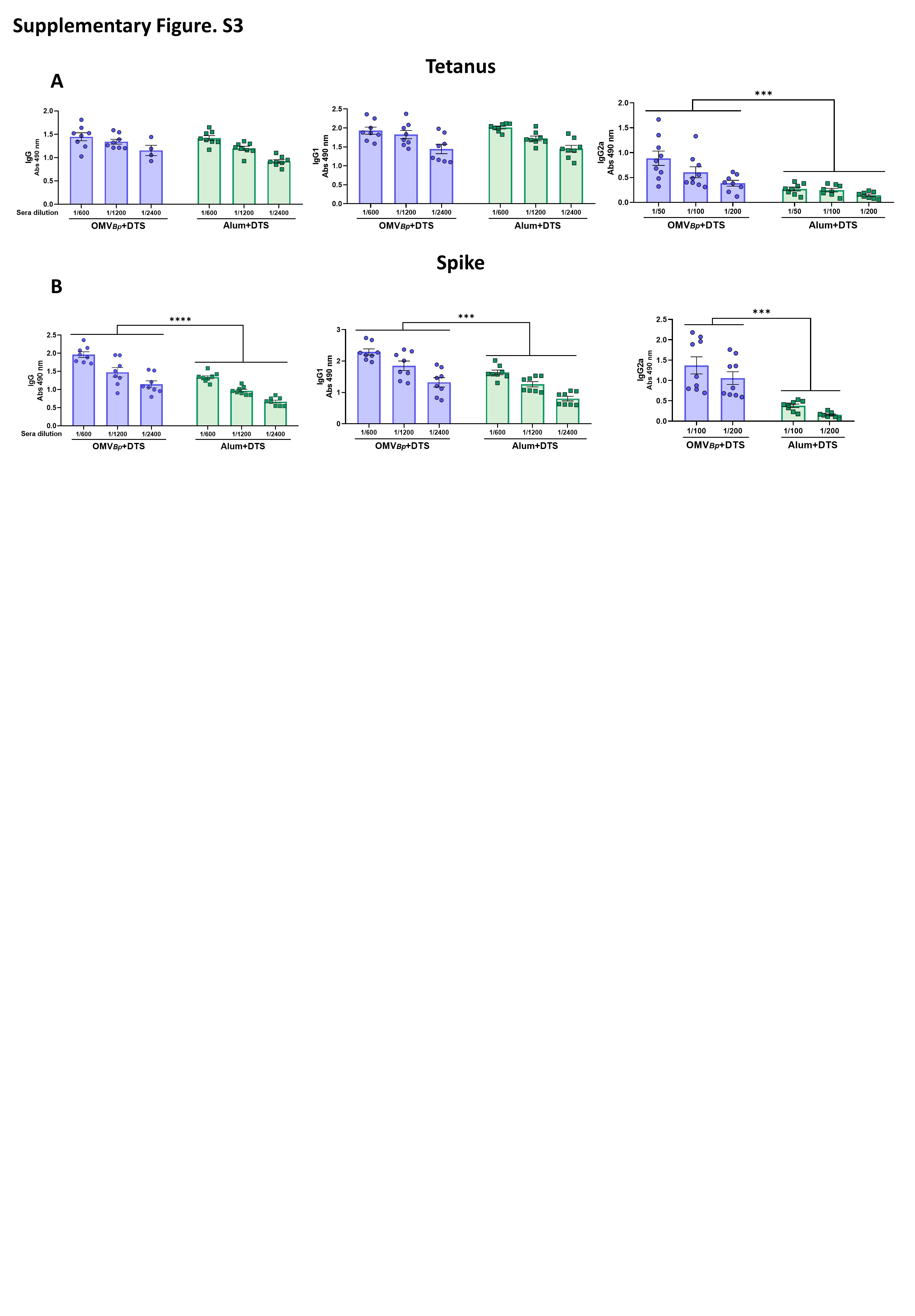

Supplement: Supplementary Figure 3 — Comparison of adjuvant properties between OMVs derived from B. pertussis and Alum. The levels of T-specific or S-specific IgG, IgG1, and IgG2a induced after the second dose of formulations containing OMV Bp or Alum as adjuvants are presented in (A, B), respectively. For these assays the quantities of the heterologous immunogens used in the formulations were the minimum amounts tested in our study (D: 0.45 μg/dose. T: 2.1 μg/dose S: 0.75 μg/dose). The levels of the different immunoglobulins were determined in sera collected 14 days after the last dose by ELISA. ****p<0.0001, ***p<0.001, by two-way ANOVA using Bonferroni for multiple comparisons. [file Image_3.jpeg]
